# The Extremely Brilliant Source storage ring of the European Synchrotron Radiation Facility

**DOI:** 10.1038/s42005-023-01195-z

**Published:** 2023-04-24

**Authors:** Pantaleo Raimondi, Chamseddine Benabderrahmane, Paul Berkvens, Jean Claude Biasci, Pawel Borowiec, Jean-Francois Bouteille, Thierry Brochard, Nicholas B. Brookes, Nicola Carmignani, Lee R. Carver, Jean-Michel Chaize, Joel Chavanne, Stefano Checchia, Yuriy Chushkin, Filippo Cianciosi, Marco Di Michiel, Rudolf Dimper, Alessandro D’Elia, Dieter Einfeld, Friederike Ewald, Laurent Farvacque, Loys Goirand, Laurent Hardy, Jorn Jacob, Laurent Jolly, Michael Krisch, Gael Le Bec, Isabelle Leconte, Simone M. Liuzzo, Cristian Maccarrone, Thierry Marchial, David Martin, Mohamed Mezouar, Christian Nevo, Thomas Perron, Eric Plouviez, Harald Reichert, Pascal Renaud, Jean-Luc Revol, Benoît Roche, Kees-Bertus Scheidt, Vincent Serriere, Francesco Sette, Jean Susini, Laura Torino, Reine Versteegen, Simon White, Federico Zontone

**Affiliations:** ESRF - The European Synchrotron, 71 Avenue des Martyrs, 38000 Grenoble, France

**Keywords:** Techniques and instrumentation, Techniques and instrumentation, Techniques and instrumentation

## Abstract

The Extremely Brilliant Source (EBS) is the experimental implementation of the novel Hybrid Multi Bend Achromat (HMBA) storage ring magnetic lattice concept, which has been realised at European Synchrotron Radiation Facility. We present its successful commissioning and first operation. We highlight the strengths of the HMBA design and compare them to the previous designs, on which most operational synchrotron X-ray sources are based. We report on the EBS storage ring’s significantly improved horizontal electron beam emittance and other key beam parameters. EBS extends the reach of synchrotron X-ray science confirming the HMBA concept for future facility upgrades and new constructions.

## Abstract (web summary)

X-ray science at modern Synchrotrons and X-ray Free Electron Laser facilities are enabling the study of subtle structural changes of matter from the single atom to the macroscopic scale. This paper reviews new concepts in synchrotron storage rings design and reports on the successful commissioning and operation of the new X-ray storage ring of the European Synchrotron Radiation Facility (ESRF) in Grenoble.

## Introduction

Modern X-ray science endeavours to provide unique tools and methods to understand the complexity of matter from the single atom to the macroscopic scale. Developing materials for cleaner energy production and storage, understanding life from proteins to working cells and organs are some of the themes at the heart of today’s challenges for human kind, and addressing them is key to construct a new sustainable world, reducing environmental impact, mitigating climate change and overcoming diseases and pandemics. In this context, the ambitious goals of 21st century science—as for example amply discussed in the UNESCO objectives of the next decade and in the Next Generation EU programmes of the European Commission—often refer to the need of understanding and visualising the hierarchical static and dynamic organisation of complex and functionalised matter with full continuity from macroscopic objects down to interactions among few atom aggregates. Some of these concepts are at the basis of the 2021 Nobel prise in physics^1^, and match precisely the objective of today’s X-ray science at modern Synchrotrons and X-ray Free Electron Laser facilities: with the aim to bridge gaps between visible light and electron microscopy and other structure of matter characterisation tools, they are enabling the study of subtle structural changes within heterogeneous atomic assemblies from ~10^12^ atoms down to only a few atoms; all of this with spatial and temporal resolutions that approaches a few atoms in space (sub-nanometre) and inter-atomic motion in time (femtosecond), respectively. Such incredible resolution and spanning range in space and time are made possible by the outstanding improvements of brilliance, spectral range and degree of spatial and temporal coherence and resolution of accelerator-based X-ray sources, which are unmatched by other structural and dynamical condensed matter characterisation tools^2^. In fact, during the last ~50 years peak brilliance has increased up to 22 orders of magnitude when compared to conventional laboratory sources.

X-ray science using synchrotron sources generated by storage rings plays a fundamental role in these advances, and nowadays in the range of 50 000 scientists worldwide use the many (~30) highly advanced third-generation sources operational around the world.

A third-generation synchrotron source is based on a storage ring with a magnetic lattice introduced by Chasman and Green (CG)^3^ in the 70’s, whose main characteristics is the minimisation and decoupling of the horizontal and vertical emittances of the stored electron (positron) beam along the whole beam path and in particular on metre-long straight sections, which are then used to produce X-rays with ad-hoc magnetic devices (undulators, whose design optimise the X-ray source emission in terms of brilliance, coherence, energy range etc.). These storage rings were truly revolutionary as they improved experimental parameters by several order of magnitude compared to previous electron/positron storage rings, which were mainly derived by the adaptation of high-energy physics colliders to the production of synchrotron radiation. They marked in fact a sharp change in design philosophy between machines optimising luminosity (small electron beam size in a collision regions of the lattice) and almost constant small beam size along the whole ring to optimise X-ray source performances and parameters.

The European Synchrotron Radiation Facility became operational with its first stored electron beam in February 1992. By demonstrating that the enormous advances promised by the CG lattice and the extensive use of undulators were indeed possible, the immediate success of the high-energy storage ring at the ESRF and similar endeavours in the US and in Japan, with the APS and SPring-8 respectively, stimulated the construction of many other third-generation source facilities worldwide during the last 30 years. They are still very successful up to this day and enabled many discoveries in fundamental science—which were recognised by numerous Nobel prises—as well as in applied and proprietary industrial science—which led to many new products, medical and industrial procedures to the benefit of human society (see ref, ^4^ and references therein).

During the construction and commissioning of the ESRF, accelerator scientists were already reflecting on how to push the CG lattice design further, with pivotal contributions from D. Einfeld et al.^5^, A. Ropert et al.^6^ on the development of the “ultimate synchrotron source” concept, and P. Elleaume and A. Ropert^7^ with the attempt to adapt these extreme theoretical designs to a possible upgrade of the ESRF lattice. However, the conclusions were not positive for the upgrade of existing facilities, as many conceptual and technological aspects did not allow an existing storage ring to be upgraded to deliver X-rays with a considerably higher brilliance whilst maintaining constant other key parameters such as electron beam energy, current, lifetime, source stability, and operational reliability. On the contrary, these concepts were successfully implemented for the construction of two new green-field projects, MAXIV in Lund-Sweden and Sirius in Campinas-Brazil. The first one has been successfully delivered in 2016^8^, and Sirius is successfully being commissioned at the time of writing.

The ESRF accelerator team, during the last decade, did not give up the hope to develop a new lattice design—which necessarily had to introduce new concepts overcoming the limitations of the CG lattice and its Multiple Bend Achromat (MBA) extrapolations, introduced by Einfeld et al. ^5^. In fact, the CG lattice is the DBA version of the MBA lattice. Their goal was to substantially improve, by a factor of 10–100, some of the key performance parameters of the X-ray sources in the initial ESRF DBA storage ring. This paper reports on the successful experimental accomplishment of this endeavour, which is based on the implementation of the Hybrid MBA concept^9,10^, and which is realised by the construction at the ESRF of the EBS storage ring. The HMBA concept enabled the required brilliance and coherent fraction to be achieved within the geometrical constraints of the existing ESRF infrastructure. The EBS, therefore, is opening a gateway to possible upgrades of all existing third-generation storage rings.

Indeed, many projects, worldwide, are at various stages of conception. Some upgrades are well advanced like the APS-U^11^ in the USA and SLS-2^12^ in Switzerland. There are also many other upgrades or new facilities being built or planned. These include the ALS-U^13^ in the USA and in Europe there are PETRA IV^14^ and BESSYIII^15^ in Germany, Elettra 2.0^16^ in Italy, Diamond II^17^ in the UK as well as ALBA II^18^ in Spain. In Japan, there is the Spring-8 II^19^ upgrade as well as SliT-J^20^ which is under construction. In China, there are HEPS^21^, WHPS^22^, Shenzhen^23^ and HALS^24^ in construction or planning. The list is undoubtedly not complete as there is a general trend in upgrading existing facilities and building new 4th generation synchrotron sources. Together with MAXIV^25^, Sirius^26^, and the ESRF-EBS^27^ there will be a formidable array of synchrotron facilities by the end of the decade.

The HMBA concept development and the EBS realisation are fundamental elements of the ESRF Upgrade Programme, which was launched by the ESRF in 2009 after a 15-year period (1994–2009) of successful user operation. Its aim is to provide new tools for X-ray science allowing us to address qualitatively better the scientific challenges outlined above. The ESRF Upgrade Programme’s key objective is to improve by several orders of magnitude (3–5) the combined effect of source and instruments upgrades, tailoring each specific X-ray instrument to its domain of application. The ESRF Upgrade Programme entails new beamline designs, detector developments, data management strategies, and most importantly the construction of a new high-energy synchrotron source with X-ray beams two orders of magnitude brighter and more coherent than those provided by the initial ESRF DBA storage ring. The HMBA lattice concept was conceived at the ESRF in 2012^28^, the EBS project was outlined immediately after in 2013, and the EBS construction launched in 2015. The commissioning and delivery of the source to the ESRF users was achieved as initially scheduled in 2017, on 25 August 2020.

In the following sections, we will describe the key innovative aspects characterising the EBS storage ring. We will cover the expected source improvements, describe the context that has led to the successful construction and commissioning of the storage ring, and present the first experimental characterisation of the EBS X-ray source and its implications for future developments in synchrotron X-ray science.

## HMBA lattice characteristics

The Hybrid Multi-bend Achromat lattice developed at the ESRF has its origins in the Multi-Bend Achromat lattice first proposed in 1995^5^. In an MBA lattice the number of dipole magnets is increased to reduce their bending angle per unit-length and consequently reduce the equilibrium emittance (see Supplementary Note 1). This idea was originally proposed by Einfeld et al. ^5^. as the natural extension of the Double-BA concept—developed by Chasman and Green in 1975^3^ and which is the basis of present third-generation synchrotron sources—and first realised in 2015 in a 3 GeV lattice, when MAXIV^8,25,29^ came into operation.

However, adapting the MBA concept to the ESRF 6 GeV accelerator complex^30^ was found to be technically *impossible*. The field strengths and gradients for the magnets were not achievable, even with state-of-the-art technology. In addition, the transverse and momentum acceptances were found to be too small to allow for accumulation of electrons using standard off-axis injection schemes^31–33^ and at the same time providing a sufficiently large beam lifetime^7,34^. It should however be noted that recent upgrade programmes have since then proposed injection schemes with very small transverse acceptance^35,36^ to overcome these limits, but these options were not considered for the new ESRF ring at the time.

In 2012, the HMBA lattice proposal introduced novel ingredients that allowed the field strengths of the magnets to be significantly reduced, while also improving the 6D acceptance of the lattice and achieving ultra-low emittances^9,10^.

**Fig. 1:**
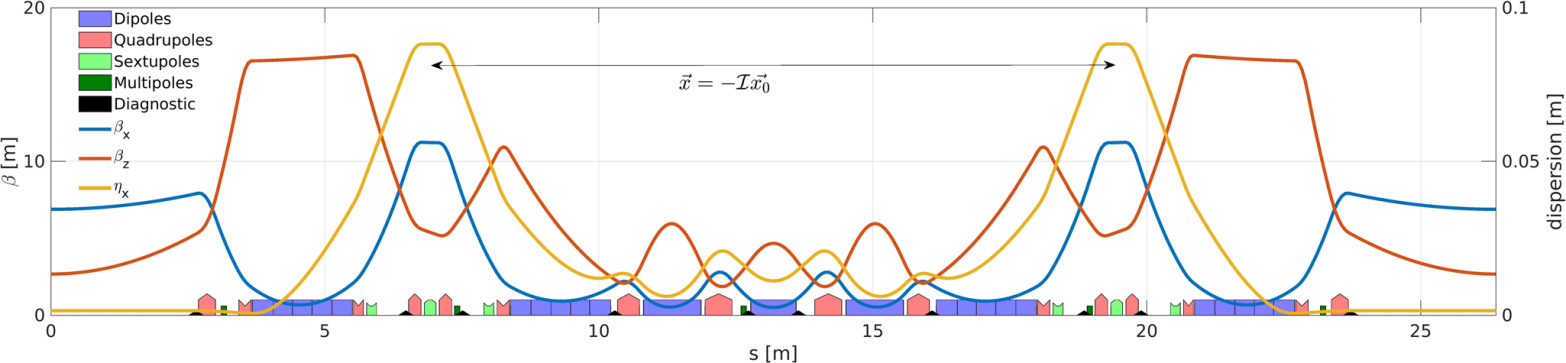
Lattice functions and magnet layout for the hybrid multi-bend Achromat SR standard cell. The Dipoles, quadrupoles, sextupoles, multipoles and diagnostic elements along the cell (position s) are shown as violet, pink, light green, dark green and black objects respectively. The *β*_*x*_, *β*_*z*_, and *η*_*x*_ parameters are shown as blue, red and orange lines respectively. Reproduced by permission^27^.

The unit cell of the ESRF HMBA lattice is shown in Fig. 1 and the main ring parameters are summarised in Table 1. Its characteristics can be summarised as follows (see also Supplementary Note 2):

- Low horizontal beta functions and dispersion at dipoles.
- Optimised sextupole layout and associated optics functions to reduce their strengths and thus magnets length. This allows for an increased number of dipoles to be accommodated in a given arc length, and also compensates for the natural chromaticity (see Supplementary Note 3) introduced by high-gradient (91 Tm^-1^) quadrupoles.
- Intrinsic compensation of non-linear resonances to provide large transverse and momentum acceptances. This allows off-axis injection with more than 90% efficiency and beam lifetime of the order of 20 h.

**Table 1:** Comparison of DBA and HMBA source parameters.

Common	6.0 GeV 844 m	32 cells	
Cell type	3rd gen.	4th gen	
	e_h_ ~ nm	e_h_ ~ 0.1 nm	
	DBA	HMBA design	
*L*_*dip*_/*L*_*total*_ *%*	18	38	Dipole filling factor
*ϵ*_*h*_ pm^.^ rad	3985	133	Horizontal emittance
*ϵ*_*v*_ pm rad	4	1	Vertical emittance
*δ*_*E*_ %	0.106	0.094	Energy spread
*b*_*l*_ mm	3.43	2.9	Bunch length
*I*_*e*−_ mA	200	200	Total beam current
*J*_*x*_	1.00	1.51	Horizontal damping partition number
*U*_0_*/turn* MeV*/*turn	4.88	2.56	Energy loss per turn
max *K*_*quad*_ Tm^−1^	16	91	Maximum quadrupole strength
max *K*_*sext*_ Tm^−2^	222	1720	Maximum sextupole strength
max *K*_*oct*_ Tm^-3^	0	36025	Maximum octupole strength
# magnets/cell	17	34	Number of magnets per cell

The table compares the synchrotron ring parameters for the former Double-Bend-Acromat (DBA) source and the new Hybrid-multi-Bend-Acromat (HMBA) source.

To achieve such overall performance parameters and overcome the technological challenge and acceptance limitations, the ESRF HMBA lattice features an improved layout for the dipoles and sextupoles as illustrated in Fig. 1. Instead of being equally distributed, the dipoles are arranged such that more space is available near the sextupoles. This choice increases the *β*-functions and dispersion at the sextupoles. As a result, their correction efficiency increases and it is possible to significantly reduce their strengths to obtain the same effective chromaticity correction (see Supplementary Note 3). In addition, the bending angle of the neighbouring permanent magnet dipoles is varied longitudinally to further increase the dispersion and slightly reduce the horizontal emittance^37,38^. Finally, the central part of the cell is composed of strong focusing quadrupoles and combined defocusing dipole-quadrupoles to optimise the space usage and increase the horizontal damping partition number *J*_*x*_ (*ϵ*_*h*_ ∝ *J*_*x*_^−1^).

Although placing the sextupoles in the high-dispersive region improves their efficiency and mitigates the negative impact of the chromaticity corrections^39^, this was still not sufficient to provide the required large acceptance for off-axis injection. A solution to this problem was found in the optics designs^40–42^ of colliders, where sextupoles are associated in pairs in order to partially cancel non-linear resonances. This principle was applied to the HMBA lattice by imposing an odd multiple of *π* phase advance, or −*I* transform^39^, between focusing sextupoles (see Supplementary Note 4).

With all these ingredients integrated into the lattice it was possible to increase the filling factor, *L*_*dip*_*/L*_*total*_, of the machine and therefore reduce the equilibrium emittance without compromising the machine performance in terms of lifetime and acceptance. Using the MAXIV^8^ 3 GeV lattice as a reference, simply scaling it to 32 cells and 6 GeV would result in sextupoles strengths of the order of 7000 T/m^2^. On the other hand, the sextupole strengths required for the HMBA lattice of the ESRF 6 GeV storage ring are much smaller as shown in Table 1. Those values are fully compatible with present technological capabilities.

The HMBA concept, therefore, provides an elegant solution to low-emittance lattice designs requiring large acceptance (100% injection efficiency in the storage ring) and a long lifetime that can be applied independently of the design energy or the ring circumference, provided that the required magnets can be manufactured.

The energy consumption of the HMBA lattice is smaller than that of previous lattice designs: the smaller radiated power *U*_0_ reduces the required Radio Frequency voltage to maintain the stored beam. Moreover, the use of permanent magnet technology for the dipoles sets their energy consumption in operation to zero.

The ESRF HMBA lattice was designed to match strict constraints imposed by the existing infrastructure, such as the storage ring tunnel length and size, the injection chain, and the number and location of the beamlines, which *de facto* imposes a 32-cell lattice. The performance presented in this section refer to the original design parameters reported in Table 1. In the future, improvements based on a growing understanding of the lattice’s detailed properties should be possible. For example, a further reduction of the photon source size by re-distributing the damping partition number and reducing the *β*-functions in straight sections, while increasing the acceptance of the machine with better matching of the optics off-energy. This and other ideas fuel ambitious ongoing Research and Development programmes at the ESRF and worldwide.

## Photon sources in the new lattice

Of the 32 straight sections of the EBS storage ring, 28 are used to produce X-ray radiation, which is then fed to the corresponding beamline, an instrument collinear with the straight section exploiting the radiation for experimental purposes. The intense X-rays in the straight section are generated using periodic magnetic arrays, and these insertion devices are called undulators (Supplementary Note 5) as they introduce a transverse undulation to the electron beam trajectory.

The main purpose of building a new storage ring is to increase the brilliance (Supplementary Note 6) of the X-ray photon beams. This section recalls how this is achieved with undulator insertion devices, the main aspects characterizing undulator radiation, and the technologies for their construction.

Brilliance is the most used figure of merit characterizing the performance of an X-ray source, as it combines the photon flux and the spatial coherence of the source: a higher brilliance translates into a higher coherent flux.

**Fig. 2:**
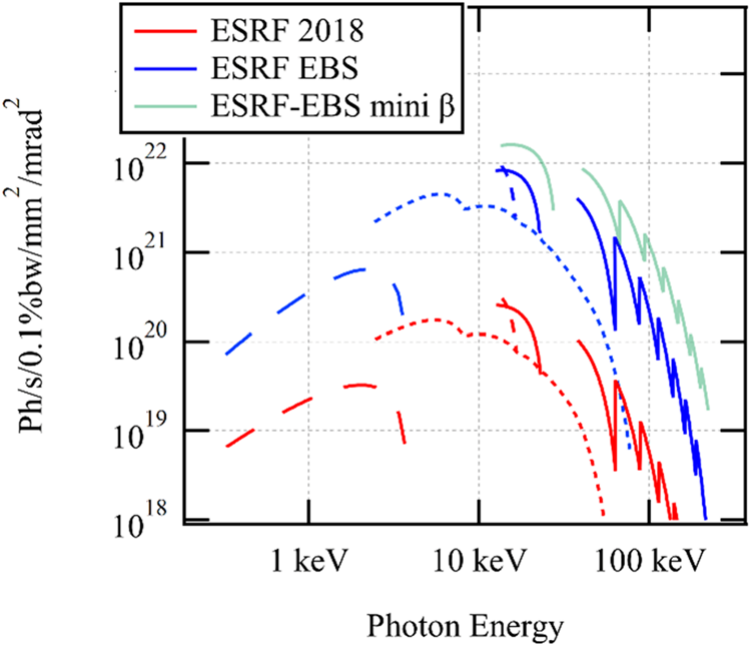
Brilliance curves. The figure shows the brilliance curves for the former ESRF lattice (red), for the EBS lattice (blue) and for a future EBS upgrade with “mini-β” sections and ultra-short-period undulators (green). The dashed curves are for ex-vacuum undulators: 88 mm period, 4 m long circular polarisation undulator at low-energy, 35 mm period, 4.8 m long planar undulator at medium energy, and 20 mm period, 4.8 m long planar undulator at 14 keV. The solid curves are for 2 m long cryogenic permanent magnet undulators (CPMUs) with 14.4 mm period for the ESRF DBA and ESRF EBS, and 12 mm (still to be built and demonstrated) for future mini-β upgrades. The brilliance curves were computed with the SRW software^125^, taking into account the energy spread and detuning^126,127^.

The brilliance is often approximated as the ratio of the photon flux to the radiation’s phase-space volume at the source location, which is the convolution of the electron beam spatial and angular sizes—proportional to the electron beam emittance in the considered transverse direction—and the X-ray source diffraction limit values. As seen in Supplementary Note 5, these are wavelength and undulator-length dependent. The HMBA lattice, with its reduced emittance, decreases dramatically the denominator at short X-ray wavelength, while the flux in the numerator is driven by the undulator technology and by the electron beam current. Figure 2 shows brilliance curves for the EBS and for the former ESRF (ESRF 2018) light source. With the same undulators, the brilliance is increased by a factor of 15 at 1 keV, 27 at 10 keV and 36 at 100 keV.

The undulator technology has been continuously improved over the last two decades, the trend being to increase the number of periods through the reduction of the magnetic gap and of the period length, which results in a larger brilliance at high-energy. The majority of the ESRF insertion devices are permanent magnet planar undulators installed in-air around the storage ring straight section vacuum chamber^43–47^. Recently, short-period in-vacuum^48–52^ and liquid-N_2_ cooled Cryo in-vacuum Permanent Magnet Undulators (PMUs and CPMUs) have been successfully developed and an increasing number of them are being installed^53–67^. Super-Conducting Undulators are also candidates for short-period undulators, and the subject of important Research and Development activities^68–74^. Other designs, based on magnetic materials (possibly high-temperature superconductors) polarised by a large solenoid, are also being studied^75–82^.

In the 10–20 keV energy range, the maximum brilliance is reached by long in-air, short-period undulators. Such undulators are almost monochromatic and not tuneable. At higher energies, it is beneficial to reduce the magnetic gap and to work with higher harmonics of the undulator spectrum: this implies to install shorter undulators around the electron beam waist, at the centre of the straight sections. The reduction of the vertical size of the electron beam by means of the so-called mini-*β* sections is being investigated. This would enable a further reduction of the minimum magnetic gap of CPMUs, thereby further improving the brilliance at high energies. Supplementary Fig. 5 shows the deflection parameter that can be reached at short periods, for some of the technologies outlined above.

## EBS storage ring commissioning and early operation results

The design, procurement, assembly, and alignment of the EBS storage ring components on its 129 girders^83^ took place in the period 2015–2018, in parallel to the continued operation of the initial ESRF storage ring. Its installation in the existing storage ring tunnel, including the dismantling of the other storage ring, took place during 2019. The EBS commissioning started in early December 2019 and achieved the design values required for user operation in early March 2020 with an effective commissioning time of ~2 months. This rapid commissioning period compares well with recent green-field accelerator projects, which typically took ~6 months^8,84,85^ to one year^86^, and was possible thanks to the knowledge and experience from the previous storage ring. The existing infrastructure was reused at a level of ~90%, and detailed preparation and testing of injectors, diagnostics, and control systems were carried out. The in-house developed accelerating Higher Order Mode damped Radio Frequency cavities, built for EBS, were pre-conditioned without beam^87,88^, and most existing insertion devices were reused, in many cases after refurbishment for improved performance.

**Fig. 3:**
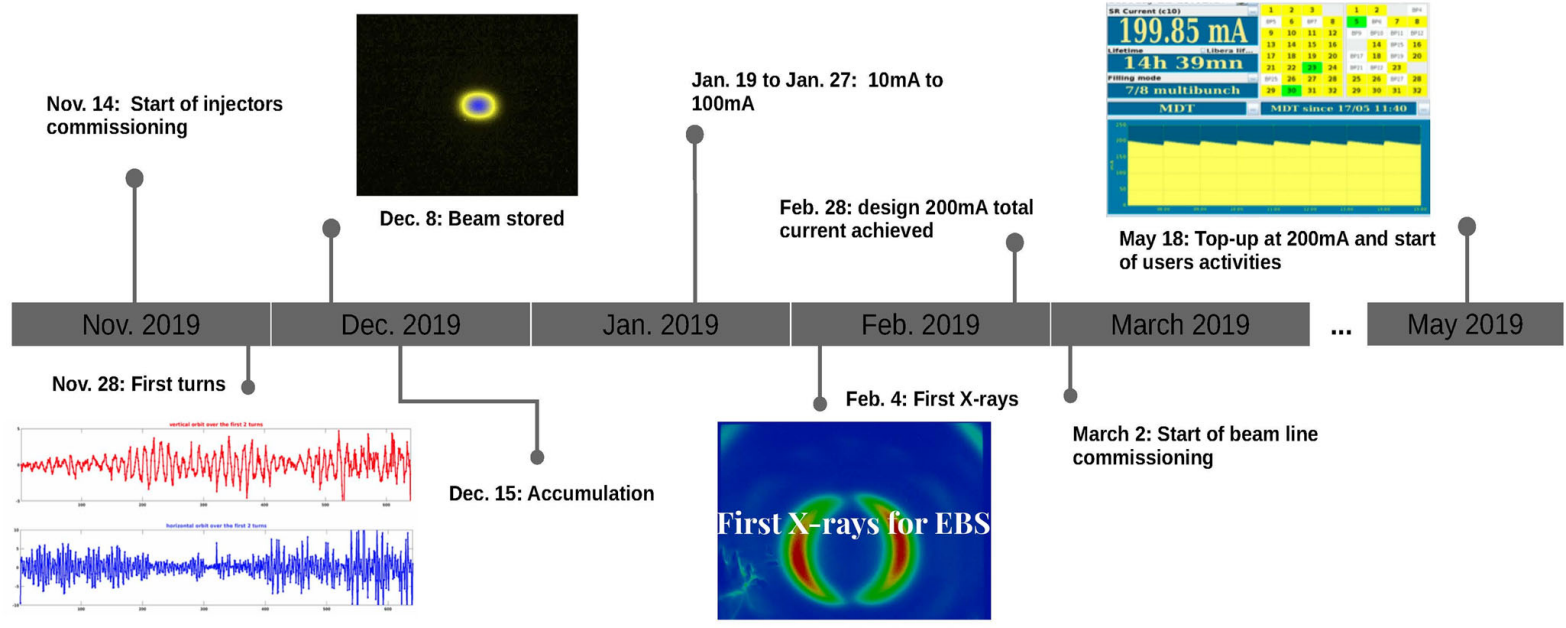
Main milestones of the ESRF-EBS machine commissioning. The figure shows the main milestones for the EBS commissioning from November 2019 when commissioning started, until May 2019 when beam was given for first user activities.

Figure 3 shows the schematic timeline of the machine commissioning and the major milestones. Details can be found in Raimondi et al. ^27^. All the goals set at the beginning of the machine commissioning period were achieved or exceeded two months ahead of schedule, enabling intensive beamline commissioning, and on-time start of operation in User Service Mode (USM) 25 August 2020 according to plan.

Table 2 summarises the design and delivered main performance parameters. The delivered values correspond to the average over one week of USM operation in uniform mode (0.2 mA per bunch and 992 bunches). In this mode, the horizontal emittance is between 120 and 130 pm rad depending on insertion device gaps setting. With full coupling correction and without external excitation, the measured vertical emittance is below 1 ± 1 pm rad. This value has a large uncertainty as it is at the limit of the resolution of the present measurement capabilities. Joint experiments with beamline experts showed only minor brilliance improvements when the vertical emittance values are below 10 pm rad, and therefore its value was set to 10 pm rad using white noise to improve beam lifetime and minimise Touschek losses. The lifetime at 10 pm rad exceeded design goals even though the machine was not yet fully conditioned and the vacuum lifetime still contributed significantly to the total value.

**Table 2:** Design goals and delivered parameters in USM condition and uniform mode.

	units	Design	Delivered	
Ie- Total beam current	mA	200	200	
Mean time between failures	h	50	>103.5	Statistics from the first two runs of 2021
Machine availability (up time percentage)	%	97	97.9	Statistics from the first two runs of 2021
Injection Efficiency	%	>90	80	
Vacuum Lifetime	h	>300	122 ± 13	All values given for *ε*_*v*_ = 10 pm rad, vacuum and Touschek lifetimes require dedicated measurements
Touschek Lifetime	h	28	41 ± 5	All values given for *ε*_*v*_ = 10 pm rad, vacuum and Touschek lifetimes require dedicated measurements
User Mode Lifetime (during machine physics runs)	h	23	>25	
*ε*_*h*_ –horizontal emittance	pm rad	140	<120 ± 8	Delivered: average with ID gaps closed
*ε*_*v*_—vertical emittance	pm rad	10	10.0 ± 0.6	Increased with white noise to optimise lifetime
Electron beam orbit stability (@20 Hz)	*σ*	0.05	<0.01	With all feedbacks operational, slow fluctuations not included

The design lifetimes were rescaled for 10 pm rad vertical emittance for comparison purpose

It is noted that although the design current was achieved early, the lifetime and average pressure were still constantly improving, consistent with the fact that the measured vacuum lifetime after one year of operation is of the order of 100 h and the design goal is 300 h.

Considering the Touschek lifetime^89^ only, which is defined by the lattice design and is the asymptotic lifetime limit for a fully conditioned machine, the present performance exceeds the design values. This is partly explained by the better-than-expected alignment of the machine magnetic lattice and the resulting smaller closed orbit (for a description of the sources of errors in real accelerators and their correction via dedicated correctors see Supplementary Notes 7 and 8).

The delivered Touschek and vacuum lifetimes correspond to measurements obtained on a fully optimised machine. However, these values in USM conditions are slightly degraded due to variations in undulator gap settings and environmental thermal drifts. In addition, the overall USM lifetime is further reduced by 5–10% due to the collimation system concentrating particle losses in dedicated shielded areas.

The value defined as the USM lifetime in Table 2 includes all these effects and represents the value delivered to the users in real running conditions.

The injection efficiency remains lower than the design value and will require further optimisation. The machine availability and mean time between failures are approaching the levels of the previous machine after only one year of operation.

The injection efficiency and *β*-beating for the bare lattice without corrections were estimated to 45% and 10–15%, respectively. These estimates were derived from measurements performed at the beginning of the commissioning with non-optimised diagnostics and tools and therefore still have large uncertainties.

The HMBA lattice is the backbone of the EBS project. Therefore, the comparison between the measured EBS parameters and the simulated ones using the HMBA reference lattice were essential to continuously optimise performances and identify errors due to magnets geometrical misalignments and magnetic fields errors (see Supplementary Notes 7 and 8). This comparison enabled both an excellent characterisation of the storage ring, and the introduction of corrections essential to reach its design properties and deliver X-ray beams with design performance.

The measured closed orbit and optics errors are at least as good as predicted, see Supplementary Table 3. The measured values correspond to the machine configuration at the end of the beam commissioning period. Due to the necessity to adjust the photon sources angles in some beamlines, dipole corrections, and close orbit have slightly increased in USM. However, the machine performance is not affected, and improved magnet alignment will allow to remove these perturbations.

Quadrupole corrections of the order of 0.2% allowed to reduce of the *β*-beating down to a few percent while the closed orbit was corrected down to 55 µm rms in both planes with horizontal and vertical dipole corrector strengths of 65 µrad and 30 µrad, respectively. The final closed orbit and dipole corrector strengths are directly related to the alignment of the machine. Smaller than expected corrections and closed orbit are therefore a clear indication that the machine alignment is better than the specified value of 50 µm rms.

The main contributions to gradient errors are feed-down effects of sextupoles introduced by a non-zero closed orbit in these magnets.

The fact that *β*-beating consistent with expectation could be achieved with smaller than-expected correction strength not only confirms the better-than-expected closed orbit but also reflects the excellent engineering work performed during the design compliance and calibration of the magnets and power supplies. As a result, most quadrupole corrections are localised in the direct vicinity of the cell sextupole magnets. This localised correction scheme combined with the intrinsic resonance compensations of the lattice provides excellent stability of the lattice and only minor adjustments related to lifetime optimisation with sextupoles, octupoles, and skew quadrupoles were required throughout the commissioning and during the first USM runs.

The EBS is a new kind of 6 GeV fourth generation light source, and as demonstrated above its delivery represents a very important step forward, also in consideration that many facilities around the world^12,14,35,36,90,91^ plan similar upgrades with similarly tight schedules.

## Measurements of the EBS source

The measurement of the brilliance and coherence of an X-ray undulator source can be carried out with direct and indirect methods. The measurement of the coherence properties of the radiation can be performed by imaging the flux at several distances from calibrated slits^92^. We estimated the brilliance indirectly from measurements of photon flux and electron beam parameters such as divergence and emittance^93,94^. The emittances of the ESRF-EBS, measured with pinhole cameras, are 120 ± 8 pm rad in the horizontal plane and 10.0 ± 0.6 pm rad in the vertical plane, as white noise is applied to improve lifetime.

**Fig. 4:**
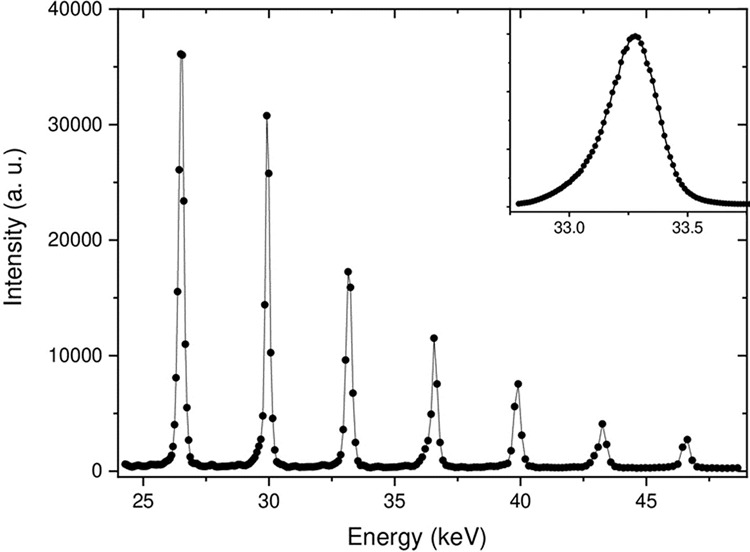
Undulator harmonics. The figure shows the 8th to 14th undulator harmonics from a 1.6 m long, U35 undulator (35 mm period) with the gap set at 12.6 mm. The inset shows a high-resolution scan of the 9th harmonic with the gap set at 13.4 mm.

Figure 4 shows an example of the first undulator spectra recorded with a silicon photodiode at 48 metres from the EBS source during the early stages of the commissioning. The source was an in-air U35 undulator with a gap set at 12.6 mm on beamline ID27. The peaks are the even and odd undulator harmonics (8th to 14th harmonic). The primary slits before the double crystal monochromator and the secondary slits after the monochromator were set to 0.7 mm (vertical and horizontal). The inset shows a fine scan of the 9th harmonic with the undulator gap set at 13.4 mm, confirming an almost symmetric peak with a narrow energy bandwidth ΔE/E of 0.5% at Full Width Half Maximum in agreement with expectations. This narrow bandpass delivered by the source is very useful for many techniques requiring the right balance between longitudinal coherence and flux versus lateral coherence (e.g. serial crystallography, Coherent Diffraction Imaging, X-ray Photon Correlation Spectroscopy, Imaging, Small Angle X-ray Scattering….) and is an important feature of the EBS source.

**Fig. 5:**
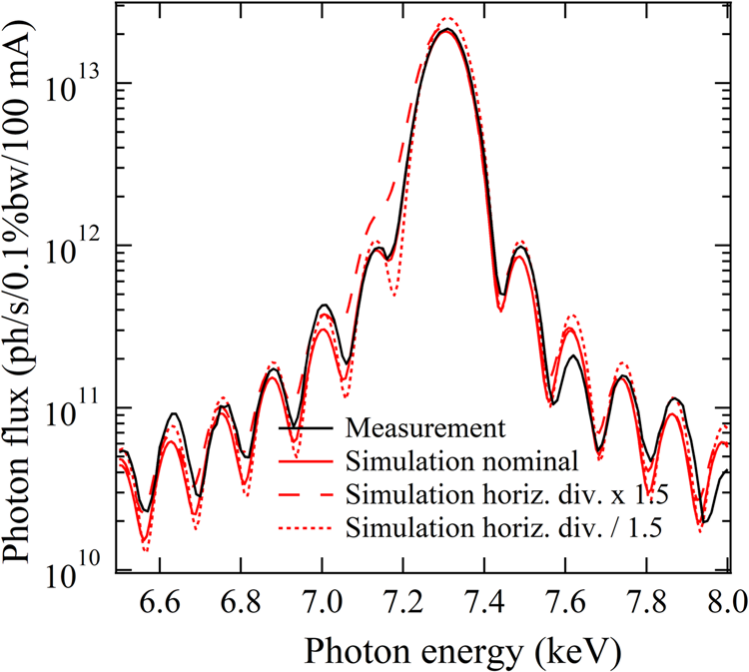
Photon flux at beamline ID10. The curves are for flux from a single 1.6 m long, 27 mm period undulator measured with a 0.15 × 0.15 mm^2^ slit at 27.2 m from the source (Measurement: black line). The undulator deflection parameter was set to 1.2, corresponding to the peak brilliance. For the simulations, the rms electron beam size was kept fixed while the electron beam divergence was set to ±50% of the nominal value (red full line, red dotted (Horizontal divergence/1.5) and red dashed (Horizontal divergence × 1.5) lines).

Figure 5 shows the comparison between the measured and calculated spectral flux of the first harmonic of an undulator installed at the beamline ID10. It demonstrates a very good agreement between the measured spectra and the computations. The fringes in this spectrum are sensitive to the divergence of the electron beam, but not to its size. Adjusting the horizontal divergence to reproduce the visibility of the first fringes on the low-energy side leads to the rms source divergence ${\sigma }_{X}{\prime} =4.2\pm 0.3$ μrad, in very good agreement with an expected value of 4.3 μrad. Combining this value with the value of the emittance, we obtain a rms value for the source size of ${\sigma }_{X}=31\pm 4$ μm.

**Fig. 6:**
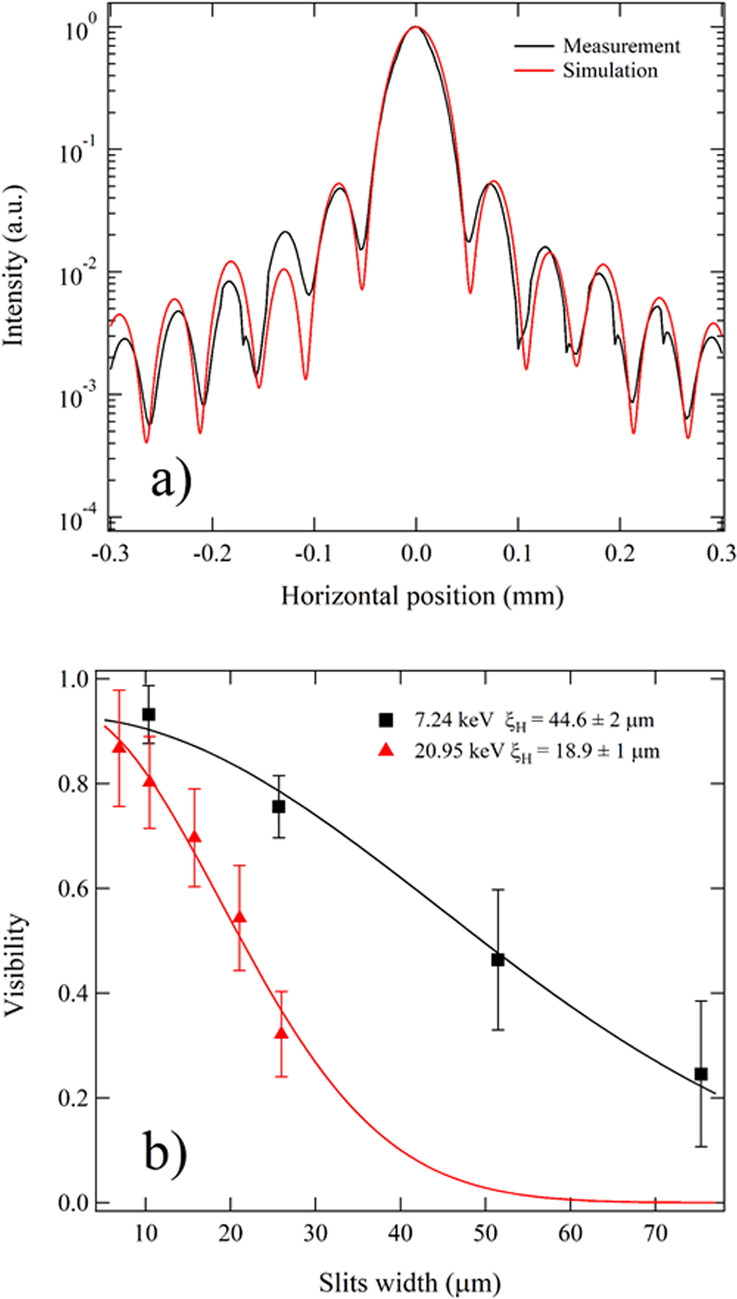
Fraunhofer diffraction patterns. **a** Fraunhofer diffraction from a single pair of slits with opening 25 × 25 μm^2^, measured (Measurement, black line) and calculated (Simulation, red line) at beamline ID10. Primary slits: 0.2 × 0.2 mm^2^ at 27 m, asymmetrical slits^95^ at 60 m and detector at 7.8 m from the slits, photon energy: 7.24 keV, detector slits 5 × 5 μm^2^. The pattern is normalised to the peak value. **b** Fringe visibility of Fraunhofer diffraction patterns as a function of the horizontal slits’ width (markers: measurements, lines: fit). The horizontal coherence length ${\xi }_{H}$ at 60 m was estimated with $V={{\exp }}(-{a}^{2}/2{{\xi }_{H}}^{2})$ were $a$ is the slit aperture^96^. The estimates for the horizontal coherence length from a Gauss-Schell model^97^ are ${\xi }_{H}=53.4$ μm at 7.24 keV and ${\xi }_{H}=19.1$ μm at 20.95 keV. The error bars are given by the he standard deviation of the visibility values for each slits setting.

Fraunhofer diffraction patterns^95,96^ were measured on the same beamline, at 60 m from the source point with slit openings ranging from 10 × 10 μm^2^ to 75 × 75 μm^2^. Figure 6a shows measured and calculated diffraction patterns for 25 × 25 μm^2^ slits. The agreement between the measured and calculated patterns is quite satisfactory. The visibility of the fringes is slightly higher in the calculated pattern, an effect which is attributed to imperfections in the beamline transfer optics.

Horizontal coherence lengths at 60 m were estimated from Fraunhofer diffraction measurements at 7.24 keV and 20.95 keV (Fig. 6b). The experimental values are in good agreement with estimates based on a Gauss-Schell Model in the incoherent source case^97^.

**Fig. 7:**
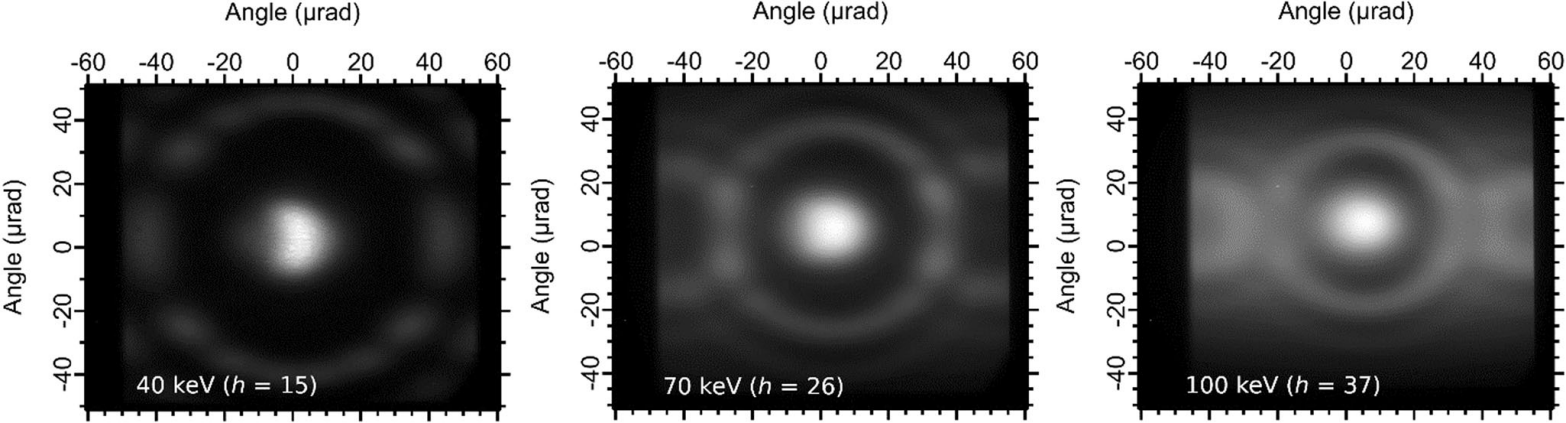
Undulator beam images. Monochromatic beam images of a 1.6 m long, U35 undulator source (35 mm period) with deflection parameter *K* = 2.293, recorded on beamline ID15A at 40.4 keV (15th harmonic), 69.9 keV (26th harmonic) and 99.5 keV (37th harmonic). The images were recorded at a distance of 65 m with a PCO-edge camera coupled to a 2× optics and a LuAG:Ce scintillator. The image pixel size is 3.18 μm pixel. The central disks are the main harmonics (e.g., the 15th harmonic at 40 keV) and the rings are higher-order harmonics (e.g., the 16th harmonic at 40 keV).

A large fraction of the ESRF insertion device beamlines operate at high X-ray energies up to 100 keV, and at these high energies the increased brightness, coherence, and angular collimation improvement of the EBS source represent significant gains. First proof of the large gain in X-ray beam performance was achieved in the early stages of the ESRF high X-ray energy beamlines commissioning with the new source. Figure 7 shows direct monochromatic photon beam images taken at beamline ID15A, which was at the time equipped with a 1.6 m long U35 in-air undulator. The X-ray beam emitted by the source passed through a crystal monochromator in Laue-Laue geometry with an energy resolution of ~0.3%, corresponding to a bandwidth slightly narrower than the undulator harmonics width.

The images recorded at 40.4 keV, 69.9 keV, and 99.5 keV with the undulator gap set to 11.1 mm show the spatial distribution of the radiation harmonics up to the highest energy and indicate a much smaller and less divergent source with respect to images seen so far at any other synchrotron source at these high X-ray energies. They confirm for the first time the complex energy- and space-dependent undulator emission pattern, which was so far broadened and hidden in a pseudo-Gaussian distribution dictated by the approximately hundred times bigger electron beam horizontal emittance.

**Fig. 8:**
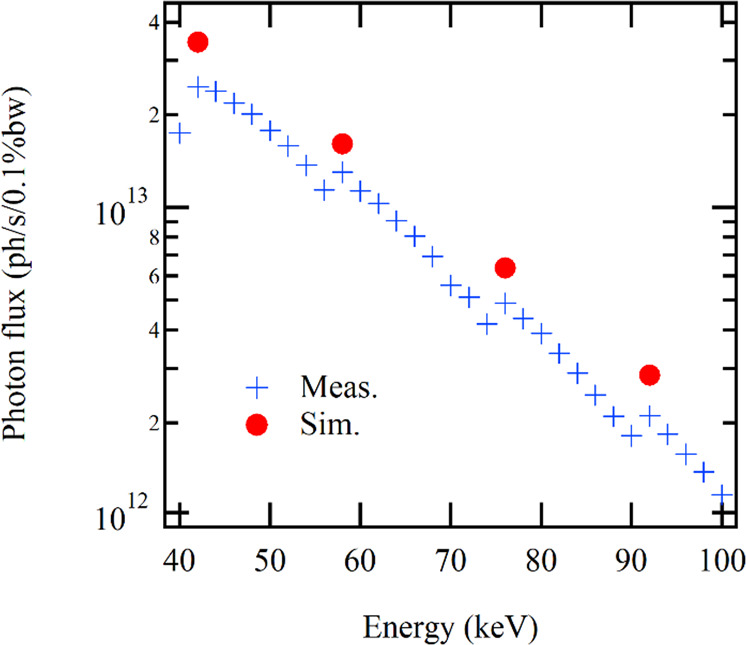
Photon flux from the 1.5 m long CPMU at beamline ID15A. The flux was measured for a 18 mm period with 0.97 T peak field at 6 mm gap, through a pair of slits set at 0.2 × 0.2 mm^2^ at 30 m distance. The plus signs represent measurements after optimising the monochromator energy for each undulator gap value. The disks are calculations for an ideal undulator (undulator harmonics 5–11). The horizontal emittance of the storage ring was 160 pm rad.

Beamlines working in the high-energy regime are now mostly equipped with CPMUs in order to maximise the available photon flux at energies up to the 100 keV range. Figure 8 shows the measured and calculated photon flux at beamline ID15A at several undulator gaps of its new CPMU18. At each gap value, the energy of the monochromator was tuned to maximise the flux through the slits. The shape of the flux curves is correctly reproduced by taking into account the spread of the undulator deflection over the periods. This spreads the synchrotron radiation along the undulator and decreases the peak flux of the higher harmonics^98–101^.

The photon measurements are in good agreement with the simulations. As it is not possible yet to measure directly the brilliance of the source, measurements of the source size and the coherent flux over a large energy range confirm that the performance of the EBS light source reaches expectations by matching simulations and theoretical expectations, as those reported in Fig. 2 and Supplementary Fig. 5.

### Outlook

The EBS represents an advance of accelerator science and engineering in the field of synchrotron radiation research. It also demonstrates the solidity and the capabilities of the HMBA design, which was invented by P. Raimondi, and developed by P. Raimondi and the ESRF Beam Dynamics Group. The EBS with its HMBA lattice is also opening up new avenues toward higher source performance and brilliance in the future. Among these options are the development of mini-beta optics, i.e. strongly increased local focussing in the straight sections where the insertion devices are localised, and the development of small-gap undulators with very short-period, which are both being pursued at the ESRF and were briefly outlined in this paper. The installation of mini-beta optics was not initially considered since it would break the symmetry of the lattice and further studies are needed to mitigate the impact on the stored beam. The main advantages are the reduction of the vertical beam size, allowing reduction of undulator gaps, and a better matching of the electron and photon beams leading to an increased brilliance. It should be noted that the mini-beta optics is suitable for short undulators only (typically 1.5–2 m).

The performance of the EBS 4th generation high-energy X-ray source is already opening new opportunities in X-ray science, and the first exciting results are being obtained. As the commissioning of the new EBS source coincided with the outbreak of the COVID-19 pandemic, the superior beam quality of the new EBS source, i.e., the much higher coherence of the X-ray beam at higher energy, was immediately exploited to support worldwide research efforts to overcome the pandemic. Biomedical imaging with hierarchical phase-contrast tomography of entire organs from the organ to the cellular level became available for the first time and is already triggering the development of a new field: 3D virtual histopathology^102,103^, which in the specific case studied at the ESRF enabled scientists to identify the virus-induced disruption and modification of the lung microvascular system^102^. There are other areas of high societal relevance where X-ray science will be increasingly useful thanks to the capabilities provided by this new generation of X-ray sources. Examples are: (1) the ability to image 3D neural networks in large volumes and with resolution down to the level of individual synapses to advance our understanding of the functioning of the brain^104^, (2) the determination of the structure of proteins when they are difficult to crystallise and to manage better beam-induced-damage, by using synchrotron serial crystallography^105–108^. This should enable the development of new drugs and new strategies in addressing disease and (3) the study of new materials for a circular economy or sustainable energy production and storage, where the need for following the relevant processes under in situ and operando conditions from the atomic to the device scale calls for new X-ray measurement protocols and approaches^109–111^. The key here is the ability to investigate composite/complex materials, such as batteries, from the scale of a single grain with the highest resolution and a wide range of contrast modalities to large assemblies of such grains/particles, as exemplified in a recent work shedding light on the chemo-mechanical behavior of Nickel-Magnesium-Cobalt cathodes by analysing the dynamics of the particle network during cycling^112^. On the more fundamental side, the availability of nanometric, high-energy, X-ray beams opens the road into unchartered territories of extreme conditions. Investigations of materials at terapascal static pressures, as they are prevalent in the larger planets of the solar system as well as in most of the exoplanets discovered so far, will become routine^113^.

The large gain in performance on the source side and the new scientific opportunities are nevertheless accompanied by a series of challenges, e.g. the need for new detector technologies to deal with the much higher data flow on many of the existing instruments and the need to provide a high-performance computing environment (hardware and software) for handling the expected massive data flow online and offline. On the other hand, the increased brilliance, coherence, and photon flux on the sample present completely new opportunities, unthinkable just ten years ago, for the development of new instruments and experimental techniques such as hard X-ray diffraction microscopy^114–117^, coherent spectroscopy^118,119^ and diffraction^114^, and phase-contrast imaging^104,120–124^ with capabilities which until now could not address a wide community as it was too close to the edge of feasibility for wide-spread and robust applications.

## Supplementary information

Supplementary Information
