# Supplementary material for: The Extremely Brilliant Source storage ring of the European Synchrotron Radiation Facility
Source: Commun Phys. 2023 Apr 24;6(1):82. doi: 10.1038/s42005-023-01195-z (PMC10124696; doi:10.1038/s42005-023-01195-z)
Supplement: Supplementary file 1 — Supplementary Information [file 42005_2023_1195_MOESM1_ESM.pdf]

Supplementary Information for

"The Extremely Brilliant Source storage ring of the European Synchrotron Radiation Facility"

Pantaleo Raimondi[1], Chamseddine Benabderrahmane[1], Paul Berkvens[1], Jean Claude Biasci[1], Pawel Borowiec[1], Jean-Francois Bouteille[1], Thierry Brochard[1], Nicholas B. Brookes[1✉], Nicola Carmignani[1], Lee R. Carver[1], Jean-Michel Chaize[1], Joel Chavanne[1], Stefano Checchia[1], Yuriy Chushkin[1], Filippo Cianciosi[1], Marco Di Michiel[1], Rudolf Dimper[1], Alessandro D'Elia[1], Dieter Einfeld[1], Friederike Ewald[1], Laurent Farvacque[1], Loys Goirand[1], Laurent Hardy[1], Jorn Jacob[1], Laurent Jolly[1], Michael Krisch[1], Gael Le Bec[1], Isabelle Leconte[1], Simone M. Liuzzo[1], Cristian Maccarrone[1], Thierry Marchial[1], David Martin[1], Mohamed Mezouar[1], Christian Nevo[1], Thomas Perron[1], Eric Plouviez[1], Harald Reichert[1], Pascal Renaud[1], Jean-Luc Revol[1], Benoît Roche[1], Kees-Bertus Scheidt[1], Vincent Serriere[1], Francesco Sette[1✉], Jean Susini[1], Laura Torino[1], Reine Versteegen[1], Simon White[1] and Federico Zontone[1]

[1] ESRF - The European Synchrotron, 71 Avenue des Martyrs, 38000, Grenoble, France

✉email: brookes@esrf.fr; sette@esrf.fr

Supplementary Note 1: Low emittance principles

The photon beam brilliance is inversely proportional to the electron beam emittance ε_x [1]:

$$\varepsilon_x = C_q \frac{\gamma^2}{J_x}\frac{I_5}{I_2} \qquad J_x = 1 - \frac{I_4}{I_2}$$

with C_q a constant, J_x the horizontal damping partition number, γ the relativistic Lorentz factor and I_n the synchrotron radiation integrals defined as follows, respectively:

$$I_4 = \oint \frac{\eta_x}{\rho}\left(\frac{1}{\rho^2} + 2k_1\right) ds \qquad I_2 = \oint \frac{1}{\rho^2} ds \qquad I_5 = \oint \frac{\mathcal{H}_x}{|\rho|^3} ds$$

ρ is the bending radius of the dipoles, k_1 the quadrupole strength and η_x the horizontal dispersion function. The integrals run over the whole lattice. $\mathcal{H}_x$ is defined by the horizontal twiss parameters β_x, α_x, and γ_x and by η_x and $\eta_{x'}$, the horizontal dispersion function and its derivative along the beam trajectory [1].

$$\mathcal{H}_x = \beta_x \eta_x'^2 + 2\alpha_x \eta_x \eta_x' + \gamma_x \eta_x^2$$

For a classic double-bend achromat [2] these equations reduce to:

$$\varepsilon_x^{DBA}[m.rad] = 5.036 * 10^{-13} E^2[GeV^2]\theta^3[\mathrm{deg}^3]$$

where E is the electron beam energy and Θ the average angle per dipole.

Low emittance can therefore be achieved with:

1) low β_x, and η_x at dipoles to reduce $\mathcal{H}_x$,

2) a large number of low-angle (θ) dipoles and

3) combined function magnets with focusing gradient k_1 and bending angle to increase J_x.

Supplementary Note 2:

Supplementary Figures 1a-c shows a comparison of different synchrotron light source lattice cells for storage rings with $E_0 = 6$ GeV and 32 cells. The figures also show the electron beam emittance (ε_x) for the different lattice cells. The characteristics of the considered lattice cells are given below.

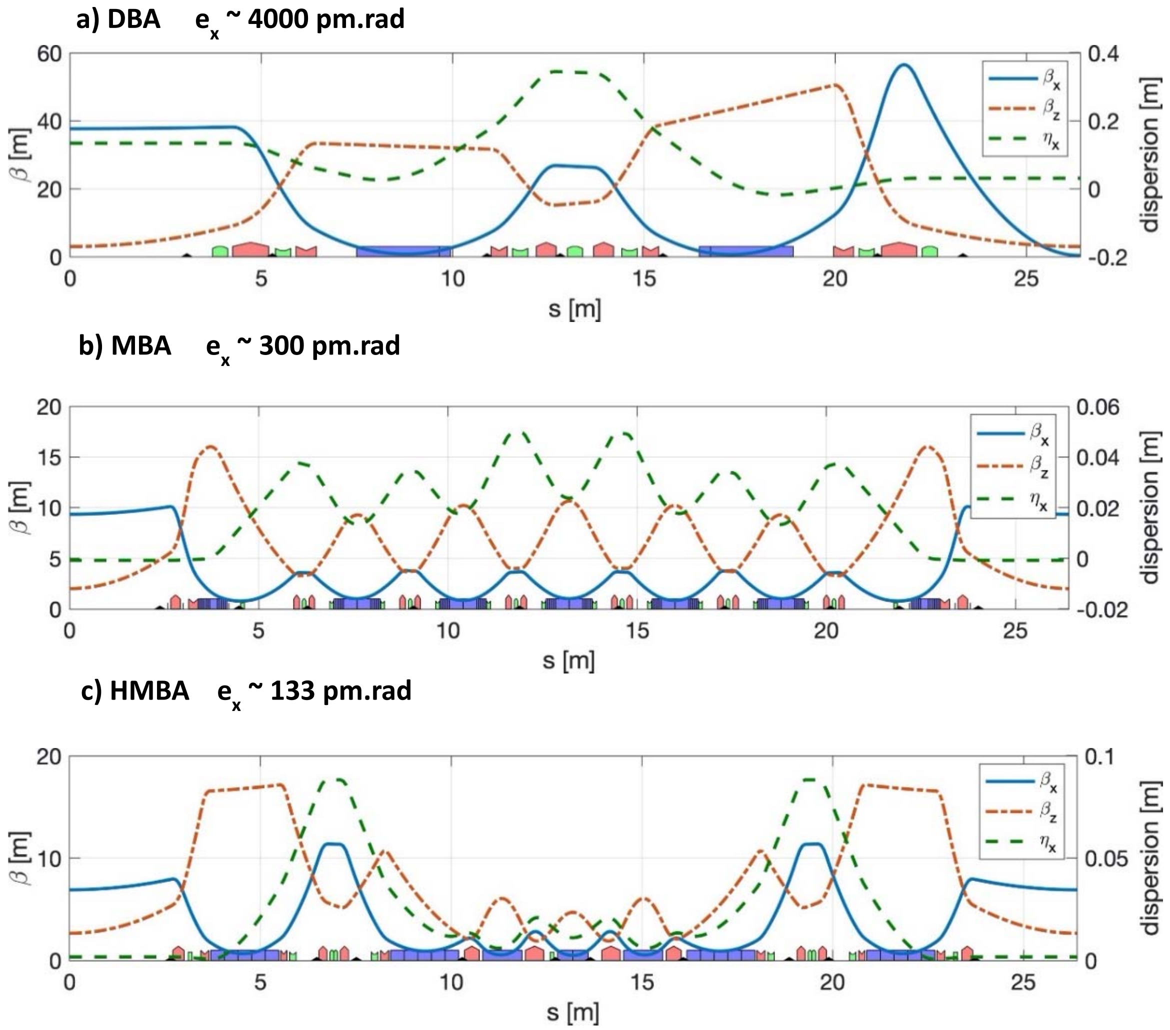

Supplementary Figure 1: comparison of different synchrotron light source lattice cells for storage rings with $E_0 = 6$ GeV and 32 cells. Supplementary Figures 1a, b and c are for the Double-Bend-Achromat (DBA), Multi-Bend-Achromat (MBA) and Hybrid-Multi-Bend-Achromat (HMBA) lattice cells, respectively.

Double-Bend-Achromat (DBA) (first light in 1992). ε_x ~ 4000 pm.rad

Characteristics:

1) small horizontal β and dispersion at dipoles (see Supplementary Note 1)

2) large dispersion at sextupoles.

Multi-Bend-Achromat (MBA) (first light in 2015). ε_x ~ 300 pm.rad

Characteristics:

1) more dipoles with less field (see Supplementary Note 1)

2) dipole-quadrupoles (J_x>1), 3) octupoles (see Supplementary Note 1).

Hybrid-Multi-Bend-Achromat (HMBA) (first light 2019). ε_x ~ 133 pm.rad

Characteristics:

1) all features of MBA

2) two large localized dispersion bumps as in DBA to increase sextupole efficiency

3) almost exact *-I* transformation (see Supplementary Note 4) between sextupoles pairs to locally cancel sextupolar aberrations

4) longitudinal gradient dipoles to increase dispersion at sextupoles

5) increased dipole filling ratio.

Supplementary Note 3: Natural chromaticity correction

Given a lattice layout (DBA, MBA, HMBA) with dipoles and quadrupoles, the sextupoles are set such that the natural chromaticity $\xi_{x,y}$ introduced by the integrated quadrupole strengths (b_2L) is neutralized or overcompensated using sextupoles with integrated strength b_3L to allow as large as possible off-momentum phase space stability and thus long lifetimes and stable high charge bunches. In this case the chromaticity is defined as

$$\xi_{x,y} = \frac{1}{4\pi}\left(\sum_{n}^{N_{sext}} 2b_3L\eta_{x,y}\beta_{x,y} - \sum_{n}^{N_{quad}} b_2L\beta_{x,y}\right),$$

where $\eta_{x,y}$ and $\beta_{x,y}$ are the horizontal and vertical dispersion and β-functions and N_{sext} and N_{quad} the number of sextupoles and quadrupoles present in the lattice. This determines the sextupole strengths, (b_3L) excluding further non-linear optimization, that are inversely proportional to the dispersion and β-functions at sextupoles. Large β-functions and dispersion at the sextupoles will therefore allow to reduce their strengths [1].

For the specific case of low emittance lattices that generally feature small dispersion (low dipole fields) and small β-functions, this can lead to strong sextupole strength, potentially reaching technological limits.

Supplementary Note 4: The $-I$ transformation between sextupoles

With b_3L the sextupole integrated strength and *x,x',y,y'* the position and angle in the horizontal and vertical planes, the electron-optical effect of a sextupole can be represented as a coordinates transformation by the transfer matrix $\mathcal{M}_s$:

$$\begin{pmatrix} x \\ x' \\ y \\ y' \end{pmatrix} = \mathcal{M}_s \begin{pmatrix} x_0 \\ x_0{}' \\ y_0 \\ y_0{}' \end{pmatrix}, \begin{pmatrix} x \\ x' \\ y \\ y' \end{pmatrix} = \begin{pmatrix} 1 & 0 & 0 & 0 \\ -0.5b_3Lx_0 & 1 & 0.5b_3Ly_0 & 0 \\ 0 & 0 & 1 & 0 \\ 0 & 0 & b_3Lx_0 & 1 \end{pmatrix} \begin{pmatrix} x_0 \\ x_0{}' \\ y_0 \\ y_0{}' \end{pmatrix},$$

If the matrix representing the region of the lattice between two consecutive sextupoles is exactly $-I$ (minus identity), as shown in supplementary Fig. 2, and we neglect the presence of other sextupoles, then

$$\mathcal{M}_t = \mathcal{M}_s(x_0, y_0).(-\mathbb{I}).\mathcal{M}_s(-x_0, y_0) = -I$$

the distortions introduced by the first sextupole at first order are cancelled by the sextupole with identical field at the other side of the *-I* region.

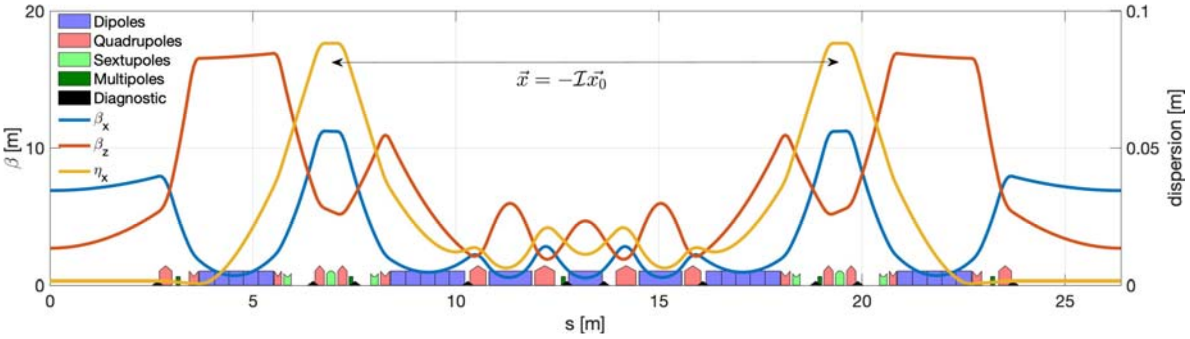

Supplementary Figure 2: Lattice functions and magnet layout for the Hybrid Multi-Bend Achromat (HMBA) Synchrotron standard cell. The Dipoles, quadrupoles, sextupoles, multipoles and diagnostic elements along the cell (position s) are shown as violet, pink, light green, dark green and black symbols respectively. The β_x, β_z, and η_x parameters are shown as blue, red and orange lines respectively. Reproduced by permission [3].

The $-I$ transformation is obtained by tuning the quadrupoles gradients such that the optics phase advance is an odd multiple of π (ϕ_x=3π, ϕ_y=π). The effect of the $-I$ transformation between sextupole pairs is shown by electron beam tracking simulation for the HMBA cell in the figure below (Supplementary Fig. 3).

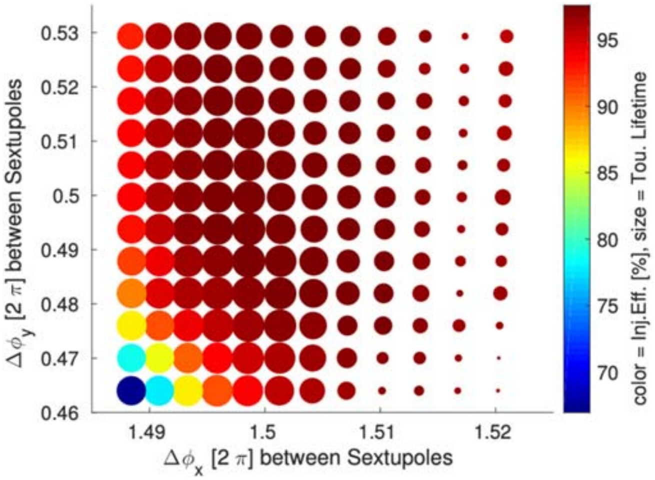

Supplementary Figure 3: electron beam tracking simulation for the HMBA cell

When moving away from the phase advances (ϕ_x=3π, ϕ_y=π) generating the $-I$ transformation, either the Touschek lifetime [4] or the injection efficiency are negatively affected. The presence of interleaved sextupoles, makes the optimum phase advances deviate slightly from the theoretical (ϕ_x=3π, ϕ_y=π).

Supplementary Note 5: Undulator radiation

Undulators create a periodic magnetic field perpendicular to the electron path, which introduces by the *Lorentz* force a periodic transverse undulation of the electron trajectory. The radiations emitted at each period interfere. This leads to a photon spectrum with narrow intense peaks. In such case, the photon spectrum shows narrow intense peaks [5, 6, 7] (harmonics) at specific photon energies. An undulator is characterised by its deflection parameter, defined as

$$K = \frac{eB_0\lambda_0}{2\pi mc}$$

where $\boldsymbol{e}$ is the elementary charge, $\boldsymbol{B_0}$ and $\boldsymbol{\lambda_0}$ are the undulator peak magnetic field and period length, $\boldsymbol{m}$ the electron mass and $\boldsymbol{c}$ the speed of light. The resonant wavelength on-axis is

$$\lambda = \frac{\lambda_0}{2\gamma^2 n}\left(1 + \frac{K^2}{2}\right)$$

$\boldsymbol{n}$ being the harmonic number and $\boldsymbol{\gamma}$ the relativistic Lorentz factor. The photon flux reaches a maximum at $\boldsymbol{K} \approx 1.2$ for the fundamental and at higher $\boldsymbol{K}$ values for higher harmonics. For a Gaussian photon beam and a single electron, the *diffraction limited* X-ray source size and divergence are respectively

$$\sigma_n = \sqrt{\lambda L/\pi} \text{ and } \sigma'_n = \sqrt{\lambda/2L}$$

$\boldsymbol{L}$ being the undulator length and λ the X-ray wavelength.

Supplementary Note 6: Spectral flux, coherence and brilliance

A single electron produces transversally coherent radiation.

However, the finite electron bunch size

$$\sigma_{x,y} = \sqrt{\epsilon_{x,y}\,\beta_{x,y} + \eta_{x,y}{}^2 \delta_E{}^2}$$

and divergence

$$\sigma'_{x,y} = \sqrt{\varepsilon_{x,y}\,\gamma_{x,y} + \eta'^2_{x,y}\,\delta_E^2}$$

δ_E being the energy spread of the electron beam, result in partially coherent radiation [8] with photon source size $\Sigma_{X,Y} = \sqrt{\sigma_n{}^2 + \sigma_{X,Y}{}^2}$ and divergence $\Sigma'_{X,Y} = \sqrt{\sigma'_n{}^2 + \sigma'_{X,Y}{}^2}$.

In practice, the radiation is coherent in the vertical plane and partially coherent in the horizontal plane, due to the ellipsoidal shape of the electron beam.

The apparent brilliance of the source, defined as $= \frac{F}{\Sigma_X \Sigma'_X \Sigma_Y \Sigma'_Y}$, combines the spectral flux F and the source sizes and divergences: high brilliance means a large number of photons in a small phase space volume, i.e. a large coherent flux. The brilliance can be interpreted as the distribution of photons in the phase space and is linked to the Wigner distribution of the electric field [9, 10, 11, 12, 13, 14, 15, 16, 17].

Supplementary Note 7: Sources of errors in storage rings

The design of a particle accelerator is based on an ideal model that assumes a perfectly aligned machine without magnetic field errors. In reality, misalignments and field errors, mostly determined by the mechanical tolerances of the magnets and the calibration of the current-to-field transfer functions, cannot be avoided. These are estimated during the design phase and the so-called design parameters represent the average of multiple seeds of errors.
We generally classify machine imperfections from the multipoles excited by such errors, and consequently the magnet type used to correct them:

- dipole errors will introduce beam displacements throughout the storage ring that can be described as follows [1]:

$$\Delta u = \sum_k M_{12}(s_m|s_k)\Theta_k$$

where Δu is the displacement of the beam at the location s_m due to many dipole-field errors Θ_k at locations s_k, and M_{12} is the transfer matrix from the perturbation s_k to the observation point s_m. The main contribution to dipole-field errors are unwanted dipole-field components, quadrupole misalignments or beam offset in quadrupole magnets.

Quadrupole-field errors will introduce a betatron tune shift and linear optics perturbations that will break the lattice properties described in the previous section and consequently degrade the lifetime, injection efficiency and emittance of the machine. The main contributors to quadrupole-field errors are unwanted quadrupole field components, sextupole misalignments or beam offset in sextupole magnets.

Reducing these errors enables the storage ring to operate closer to the ideal model, and thus improve performance. The strength of the corrections applied to bring the machine towards its ideal model is therefore a good indicator of how well the components have been manufactured and the storage ring assembled.

Supplementary Note 8: Orbit and optics correction

Good control of the closed orbit and linear optics are essential to achieve the design performance of the accelerator. The closed orbit is measured using beam position monitors and then minimized using dipole correctors. To do so, Single Value Decomposition inversion [18] is used to minimize to sum of the squares of the orbit distortions at the beam position monitors [1] :

$$(u_m - \Delta u_m)^2_{min} = (u_m - M\ \Theta_n)^2_{min}$$

where u_m is the measured position at monitor m, M is the orbit response matrix of all the correctors n and Θ_n are the vectors of the strength applied to the dipole correctors to minimize perturbations. Similarly, linear optics are corrected using quadrupole correctors by inverting a matrix of the beam response to quadrupole perturbations.

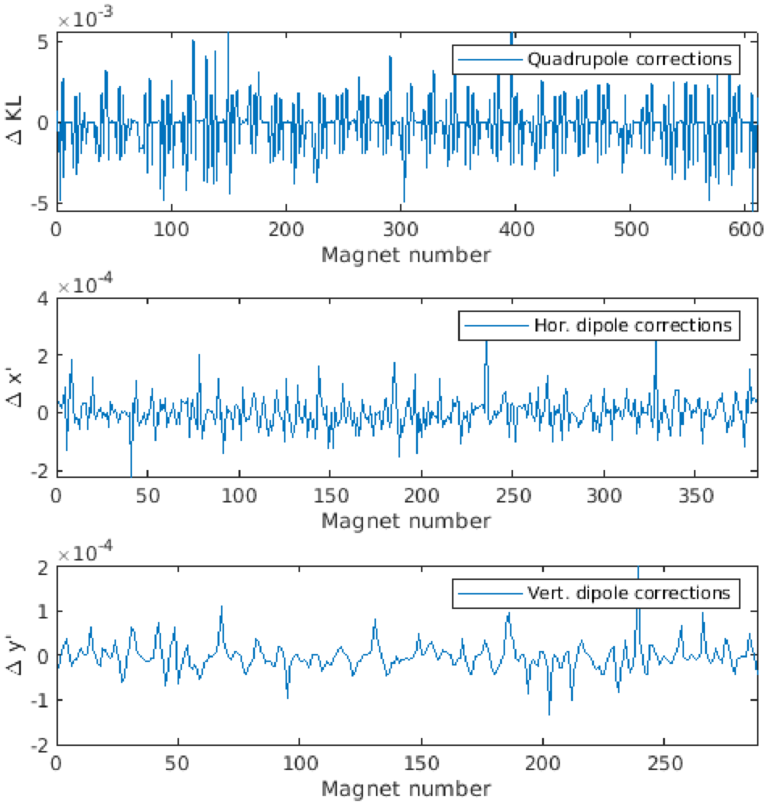

Supplementary Figure 4: Magnetic corrections applied to ESRF-EBS storage ring.

The figure shows the magnetic corrections applied to the ESRF-EBS storage ring. All corrections are below the expected values from the error model as shown in Supplementary Table 3.

Supplementary Table 3:

Measured and model rms corrections, closed orbit and optics errors.

	unit	Measured	Model
Gradient corr.	[m^{-1}]	1.7 10^{-3}	2.6 10^{-3}
Dipole corr. [H/V]	µrad	65/30	160/120
β-beating	%	1-2 ±2	1
Closed orbit [H/V]	µm	55/55	140/80

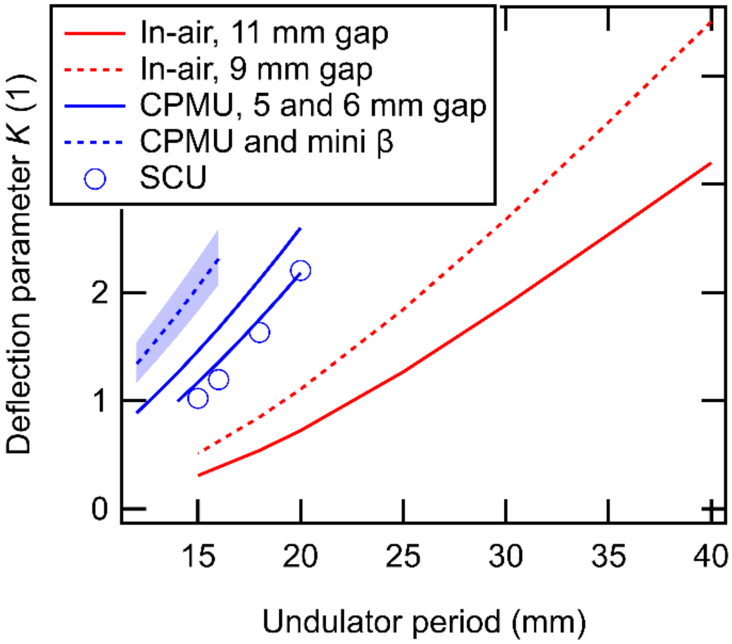

Supplementary Figure 5: Undulator deflection parameter versus period, for different undulator technologies. In a first approximation, the peak magnetic field varies as $B \propto Exp((-\pi g) / \lambda_0)$, where g is the magnetic gap. The parameter K varies with λ_0, resulting in small deflections, limited flux and poor tunability at short periods. At the ESRF, most of the undulators are installed around a 10 mm vacuum chamber with a minimum gap of 11 mm. Prototype vacuum chambers with 8 mm thickness will be installed for tests, allowing the installation of undulators with a gap of 9 mm. The CPMUs are operated at gaps in the 5 to 6 mm range, depending on their length. Operated at 77 K in most cases, they compete with Super-Conducting Undulators operated at helium temperature due to their smaller magnetic gaps. Reducing the vertical size of the beam by means of a mini-β setup would allow to install devices with ultra-small gap. The minimum gap of such devices is not determined yet but is expected to be at least in the 3.5 to 4 mm range.

Supplementary References

1. Wiedemann, H., *Particle Accelerator Physics* (Springer Berlin Heidelberg, 2007).

2. Chao, A. W., Mess, K. H., Tigner, M. & Zimmermann, F., *Handbook of Accelerator Physics and Engineering* (WORLD SCIENTIFIC, Copenhagen, 2012).

3. Raimondi, P. *et al.*, Commissioning of the hybrid multibend achromat lattice at the European Synchrotron Radiation Facility. *Physical Review Accelerators and Beams* **24**, 110701 (2021).

4. Bernardini, C. *et al.*, Lifetime and Beam Size in a Storage Ring. *Physical Review Letters* **10**, 407–409 (1963).

5. Walker, R. P., Insertion Devices: Undulators and Wigglers. *CERN Accelerator School on Synchrotron Radiation and Free Electron Laser.* (1996).

6. Elleaume, P. *Undulator, Wigglers and their applications, H. Onuki and P. Elleaume, Editors. 2003, Taylor & Francis.*, 69-107 (2003).

7. Clarke, J. A., *The Science and Technology of Undulators and Wigglers* (Oxford University Press, 2004).

8. Vartanyants, I. A. & Singer, A., Coherence properties of hard x-ray synchrotron sources and x-ray free-electron lasers. *New Journal of Physics* **12**, 035004 (2010).

9. Nash, B. *et al.*, Detailed x-ray brightness calculations in the sirepo GUI for SRW. *AIP Conference Proceedings* **2054**, 060080 (2019).

10. Nash, B. *et al.*, Undulator radiation brightness calculations in the Sirepo GUI for SRW. *Advances in Laboratory-based X-Ray Sources, Optics, and Applications VII* **11110K** (2019).

11. Bazarov, I. V., Synchrotron radiation representation in phase space. *Physical Review Special Topics - Accelerators and Beams* **15**, 050703 (2012).

12. Kim, K.-J., Brightness, coherence and propagation characteristics of synchrotron radiation. *Nuclear Instruments and Methods in Physics Research Section A: Accelerators, Spectrometers, Detectors and Associated Equipment* **246**, 71–76 (1986).

13. Geloni, G., Kocharyan, V. & Saldin, E., Brightness of synchrotron radiation from wigglers. *Nuclear Instruments and Methods in Physics Research Section A: Accelerators, Spectrometers, Detectors and Associated Equipment* **807**, 13–29 (2016).

14. Geloni, G., Kocharyan, V. & Saldin, E., Brightness of synchrotron radiation from undulators and bending magnets. *Journal of synchrotron radiation* **22**, 288-316 (2015).

15. Geloni, G., Serkez, S., Khubbutdinov, R., Kocharyan, V. & Saldin, E., Effects of energy spread on brightness and coherence of undulator sources. *Journal of Synchrotron Radiation* **25**, 1335–1345 (2018).

16. Tanaka, T. & Kitamura, H., Universal function for the brilliance of undulator radiation considering the energy spread effect. *Journal of Synchrotron Radiation* **16**, 380–386 (2009).

17. Walker, R. P., Undulator radiation brightness and coherence near the diffraction limit. *Physical Review Accelerators and Beams* **22**, 328–337 (2019).

18. Y. Chung & K. Evans, J., Closed Orbit Correction Using Singular Value Decomposition of the Response Matrix. *Proceedings of the 1993 Particle Accelerator Conference 1993, in Washington, D.C*, 2263-2265 (1993).
